# Gas Plasma-Conditioned Ringer’s Lactate Enhances the Cytotoxic Activity of Cisplatin and Gemcitabine in Pancreatic Cancer In Vitro and In Ovo

**DOI:** 10.3390/cancers12010123

**Published:** 2020-01-02

**Authors:** Kim-Rouven Liedtke, Eric Freund, Maraike Hermes, Stefan Oswald, Claus-Dieter Heidecke, Lars-Ivo Partecke, Sander Bekeschus

**Affiliations:** 1 Department of General, Visceral, Thoracic and Vascular Surgery, Greifswald University Medical Center, 17475 Greifswald, Germany; Kim.Liedtke@med.uni-greifswald.de (K.-R.L.); eric.freund@inp-greifswald.de (E.F.); maraike.hermes@stud.uni-greifswald.de (M.H.); Claus-Dieter.Heidecke@med.uni-greifswald.de (C.-D.H.); Ivo.Partecke@med.uni-greifswald.de (L.-I.P.); 2 Centre for Innovation Competence (ZIK) *plasmatis*, Leibniz Institute for Plasma Science and Technology (INP Greifswald), 17489 Greifswald, Germany; 3 Department of Clinical Pharmacology, Greifswald University Medical Center, 17475 Greifswald, Germany; stefan.oswald@uni-greifswald.de; 4 General-, Visceral-, and Thoracic Surgery, Helios Clinic Schleswig, 24837 Schleswig, Germany

**Keywords:** anticancer drugs, combination therapy, kINPen, plasma medicine, reactive oxygen and nitrogen species, ROS

## Abstract

Pancreatic cancer is one of the most aggressive tumor entities. Diffuse metastatic infiltration of vessels and the peritoneum restricts curative surgery. Standard chemotherapy protocols include the cytostatic drug gemcitabine with limited efficacy at considerable toxicity. In search of a more effective and less toxic treatment modality, we tested in human pancreatic cancer cells (MiaPaca and PaTuS) a novel combination therapy consisting of cytostatic drugs (gemcitabine or cisplatin) and gas plasma-conditioned Ringer’s lactate that acts via reactive oxygen species. A decrease in metabolic activity and viability, change in morphology, and cell cycle arrest was observed in vitro. The combination treatment was found to be additively toxic. The findings were validated utilizing an in ovo tumor model of solid pancreatic tumors growing on the chorion-allantois membrane of fertilized chicken eggs (TUM-CAM). The combination of the drugs (especially cisplatin) with the plasma-conditioned liquid significantly enhanced the anti-cancer effects, resulting in the induction of cell death, cell cycle arrest, and inhibition of cell growth with both of the cell lines tested. In conclusion, our novel combination approach may be a promising new avenue to increase the tolerability and efficacy of locally applied chemotherapeutic in diffuse metastatic peritoneal carcinomatosis of the pancreas.

## 1. Introduction

With approximately 18.1 million new cases diagnosed and about 9.6 million deaths in 2018, cancer is one of the most significant global medical challenges of our time [1]. It represents the most common cause of death in the western world within the younger population below the age of 80 [2]. Numerous improvements in the diagnosis and treatment of cancer, notwithstanding, pancreatic cancer (PC) remains one of the most lethal cancers with an almost equally incidence and mortality [3]. Due to non-specific symptoms in the early stages of the disease, the diagnosis is often made late in patients already suffering from advanced metastatic disease [4]. Even with advanced surgery (e.g., vessel reconstruction and neoadjuvant chemotherapy), only about a quarter of patients are suitable for curative surgery [5,6]. With maximal surgery and adjuvant chemotherapy (chemotherapy = CTx), the five-year survival rate is nonetheless unsatisfactorily low with 17.5–28.8% [7,8]. In addition, the incidence of PC is on the rise, and by 2030, it is expected to be the second most common cause of cancer-related deaths [9].

Due to the unsatisfactory clinical results, new therapeutic approaches are urgently needed. The combination of chemotherapeutics with reactive oxygen species (ROS) or ROS-producing drugs has long been debated as an exciting approach in cancer therapy [10]. Among the technical approach to locally generate ROS is photodynamic therapy, mainly generating singlet oxygen [11], and laser treatment of pancreatic cancer. The laser treatment does produce significant anticancer effects; however, it is unpractical in its clinical application [12]. Another option to generate multiple ROS is the application of cold physical plasma. Not to be confused with blood plasma, gas plasmas are electrically neutral gases that can be generated at tissue-compatible temperatures of about 40 °C. As a result of the (partial) ionization, ambient air serves as a reservoir to generate vast amounts of ROS [13].

Several groups, including us, have previously demonstrated anti-tumor effects with physical plasma treatment [14,15,16]. Some studies already highlighted an added value when combining direct plasma treatment with chemotherapeutic [17,18,19,20]. Apart from the possibility of directly treating cells or tissues, significant efforts have also been undertaken to utilize the therapeutic capacity of liquids exposed to gas plasmas [21]. While initially, cell culture medium was used predominantly for this purpose in vitro and even in vivo [22,23], it became increasingly clear that only clinically relevant liquids such as sodium chloride and Ringer’s lactate harbor the translational potential to improve therapeutic outcomes in preclinical and clinical models [24]. Ringer’s lactate solution or liquid showed to be especially promising candidates because central mediators such as hydrogen peroxide (H_2_O_2_) are stable over weeks while the lactate serves as an essential bystander for the anti-cancer effects observed [25].

Most standard cancer therapies involved one or several oncological treatments based on decades of clinical experience, i.e., surgery, radiation, immunotherapy/targeted therapy, and chemotherapy. With ever more combination therapies emerging at the clinical horizon, it seems clear that the value of plasma-conditioned liquids likely is to serve as an additive compound to existing therapies. One example is the hyperthermic intraperitoneal chemotherapy (HIPEC), where a chemotherapeutic agent dissolved into a liquid is perfused in the peritoneal cavity to locally attack metastatic tumor nodes in conjunction with cytoreductive surgery [26]. There is a great need for increasing efficacy and decreasing the side effects of this therapy based on the drugs commonly used [27]. To this end, we investigated the combined effect of plasma-conditioned liquid and the HIPEC drugs cisplatin and gemcitabine in pancreatic cancer cells in vitro (two-dimensional monolayers) and in ovo (three-dimensional tumor with blood supply and matrix remodeling). Promising additive tumor-toxicity was observed that might optimize intraperitoneal perfusion in future therapies of pancreatic cancers or peritoneal carcinomatosis by reducing drug concentrations and thereby decreasing side effects while maintaining a similar efficacy.

## 2. Results

### 2.1. Cisplatin, Gemcitabine, and Plasma-Conditioned Ringer’s Lactate Inactivated Pancreatic Cancer Cells in a Dose-Dependent Manner

**Figure 1:**
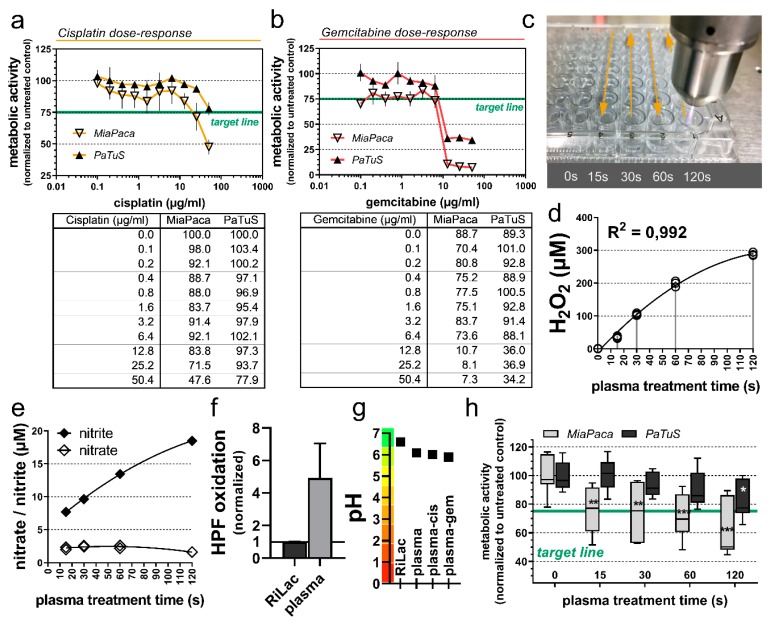
Dose-depended inactivation of pancreatic cancer cells through the application of cisplatin, gemcitabine, or physical plasma-conditioned Ringer’s lactate. (**a**,**b**) the metabolic activity of MiaPaca and PaTuS pancreatic cancer cells 24 h post-exposure to (**a**) cisplatin and (**b**) gemcitabine IC-25 target-line (green); (**c**) schematic overview of the standardized exposure of 100 µL Ringer’s lactate to the effluent of a kINPen argon plasma jet for 0, 15, 30, 60, and 120 s, and (**d**) the amount of hydrogen peroxide (H_2_O_2_) generated in this liquid; (**e**) metabolic activity of MiaPaca and PaTuS pancreatic cancer cells 24 h post-exposure to the respective physical plasma-conditioned Ringer’s lactate. Data are (**a**,**b**) mean ± SD and are representatives out of three independent experiments, (**d**) individual technical replicates with curve fitting of a quadratic function (R^2^ = 0.992) from three technical replicates; (**e**) concentration of nitrate and nitrite in plasma-conditioned liquid; (**f**) oxidation of hydroxyphenyl fluorescein (HPF) in Ringer’s lactate through 120 s of plasma-treatment; (**g**) pH value of 120 s plasma-conditioned Ringer’s lactate and plasma in combination with cisplatin and gemcitabine; (**h**) metabolic activity of pancreatic cancer cells with IC-25 target-line (green) 24 h post-exposure to physical plasma-conditioned Ringer’s lactate as representative out of three independent experiments showing median and min to max. Statistical significance was calculated utilizing ANOVA.

To test the effect of cisplatin or gemcitabine in combination with physical plasma-conditioned *Ringer’s* lactate (RiLac), the first step was to identify the optimal concentration of each compound to reach a 25% reduction in the cancer cells metabolic activity (IC-25). The metabolic activity of the cells is analyzed utilizing their capacity to reduce resazurin to the fluorescent resorufin. The transformation of this metabolite correlates with the cells’ metabolic activity, so it can be used to describe the percentage of cancer cells that were inactivated through the different treatment regimens [28]. Cisplatin inactivated MiaPaca and PaTuS human pancreatic cancer cells in a dose-dependent manner (Figure 1a). The PaTuS cancer cells were more resistant to both drugs, and to reach the IC-25, 50 µM of cisplatin were needed, while a similar effect was reached with 25 µM of gemcitabine. The toxic effect of gemcitabine was detectable at lower concentrations (Figure 1b). As the next step, an oxidative liquid was generated using cold physical plasma (Figure 1c) using Ringer’s lactate (RiLac). RiLac is a well known and clinically applied liquid and could already proof in a previous study to be an excellent candidate to store physical plasma-derived oxidants [24]. The idea was to combine this liquid with chemotherapy in two ways. The oxidative liquid was applied before chemotherapy to investigate sensitization to chemotherapy with oxidative stress (plasma-CTx), or chemotherapy was added first to sensitize cells to oxidative stress (CTx-plasma). One crucial mediator of the effect of plasma-conditioned liquid is hydrogen peroxide (H_2_O_2_) that was deposited in RiLac through the exposure to the effluent of the kINPen argon-plasma jet in a treatment time-dependent fashion (Figure 1d). Treatment times of 60 s generated 200 µM of H_2_O_2_ while 120 s generated approximately 300 µM. To calculate reliable values of generated hydrogen peroxide, the amount of evaporated liquid during these long plasma treatment times was supplemented with double-distilled water (Supplementary Figure S1a). Besides H_2_O_2,_ the time-dependent deposition of nitrate and to a greater extent nitrite was detected in plasma-conditioned RiLac (Figure 1e). The oxidational capacity of the plasma-conditioned RiLac was validated via oxidation of the hydroxyl radical and peroxynitrite anion indicator hydroxyphenyl fluorescein (HPF, Figure 1f). Ringer’s lactate is an ideal oxidative solution, it lacks buffer capacity and long plasma-treatment times could induce a drop in the pH level of the liquid. However, 120 of the exposure of RiLac to gas plasma only modest decreased the pH, which was also the case for a combinational regimen with cisplatin and gemcitabine (Figure 1g). MiaPaca cells reached the 25% reduction of their metabolic activity after exposure to 60 s plasma-conditioned RiLac, while PaTuS needed 120 s (Figure 1e) to reach the target IC-25. These experiments defined the treatment modalities that were kept constant throughout all further experiments in this study (Table 1).

### 2.2. Combination of Cisplatin and Gemcitabine with Physical Plasma-Conditioned Ringer’s Lactate Enhanced the Inactivation of Pancreatic Cancer Cells

**Figure 2:**
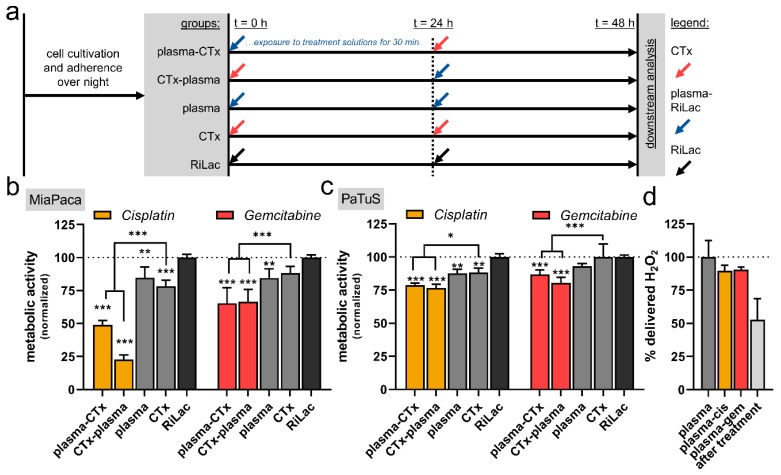
Additive decrease of metabolic activity using cisplatin or gemcitabine with physical plasma-conditioned Ringer’s lactate in pancreatic cancer cells. (**a**) Experimental procedure of the in vitro treatment regimens with the application of different treatment liquids (30 min exposure time; CTx = chemotherapeutic agents, plasma = physical-plasma-conditioned Ringer’s lactate, RiLac = Ringer’s lactate before the solutions were replaced with fresh cell culture medium) at 0 h and 24 h, as well as downstream analysis at 48 h; (**b**,**c**) the metabolic activity at 48 h of (**b**) MiaPaca and (**c**) PaTuS pancreatic cancer cells; (**d**) percent of the amount of H_2_O_2_ initially induced through the plasma treatment in the combinational regimen and post-exposure to plasma-conditioned RiLac. Data (**b**,**c**) are representatives out of eight independent experiments, or (**d**) representatives showing mean +SD. Statistical significance was calculated utilizing ANOVA.

To next identify the benefit of combination therapy, cisplatin or gemcitabine were combined with physical plasma-conditioned RiLac and compared to the respective single treatment regimens. For this, cells were exposed to one CTx, plasma-RiLac, or RiLac alone for 30 min before the respective liquid was removed and exchanged with the cell culture medium. After 24 h of culture, the respective second treatment was performed, and the cells were now exposed to the complementary treatment as foreseen in the combination therapy regimen (Figure 2a). The downstream analysis of the cells was performed 24 h after the addition of the second treatment or control condition (i.e., 48 h after exposure to the first treatment or control condition). Plasma-condition RiLac and CTx alone showed a modest but significant reduction of the metabolic activity in MiaPaca (Figure 2b) but not PaTuS (Figure 2c) pancreatic cancer cells. In contrast to these results, the combination of gemcitabine and cisplatin with plasma-RiLac (in both plasma-CTx and CTx-plasma treatment protocols) induced a significant inactivation of PaTuS cells (Figure 2c) with cisplatin having a stronger combination effect compared to gemcitabine. This was also observed in MiaPaca cells that, in general, responded stronger to combination treatment with CTX and plasma-RiLac (Figure 2b) as compared to the other regimens and PaTuS cells, respectively. The concentration of hydrogen peroxide was measured in the combinational regimen, showing only less difference to the mono treatment with plasma-conditioned RiLac, and also in wells that received fresh plasma-RiLac 30 min before (Figure 2d). This showed a 50% decrease in H_2_O_2_ through the reaction with the cancer cells. In order to gain more knowledge about the vital role of H_2_O_2_ in mediating the cytotoxic effect experiments were carried out were H_2_O_2_ was supplemented to RiLac in the same amount generated through the plasma-treatment. The combinational regimen, containing H_2_O_2_ only, also diminished the cancer cells’ metabolic activity but was less effective (Supplementary Figure S1b,c). In further control experiments, the H_2_O_2_-scavenging enzyme catalase was added to all treatment regimens and completely diminished the effect of plasma-conditioned RiLac, validating the critical role of H_2_O_2_ for the plasma effect (Supplementary Figure S1d–g). Interestingly, the scavenging could not fully prevent the effect when plasma-conditioned RiLac was applied in combination with CTx.

### 2.3. Combination of Cisplatin and Gemcitabine with Physical Plasma-Conditioned Ringer’s Lactate Mediated Terminal Cell Death to Pancreatic Cancer Cells

**Figure 3:**
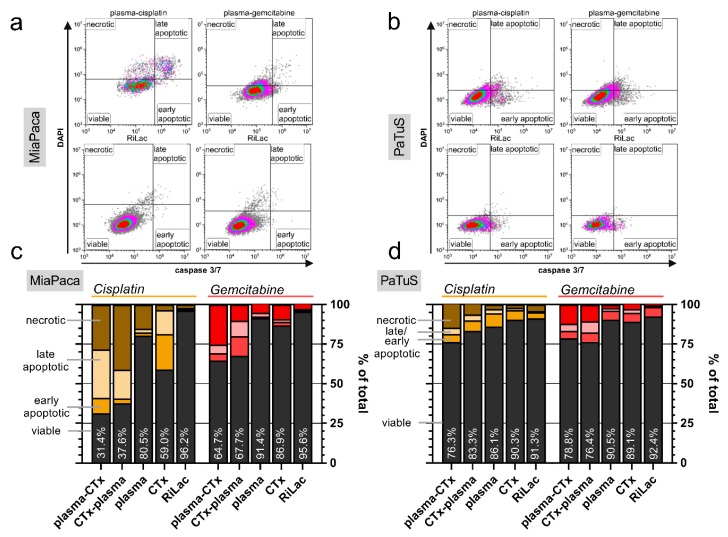
Combination of cisplatin and gemcitabine with physical plasma-conditioned Ringer’s lactate induced toxicity in pancreatic cancer cells. (**a**,**b**) representative gating of viable (DAPI^-^, caspase^-^), early apoptotic (DAPI^-^, caspase^+^), late apoptotic (DAPI^+^, caspase^+^) and necrotic (DAPI^+^, caspase^-^) MiaPaca (**a**) or PaTuS (**b**) cells at 48 h after exposure to plasma-CTx, CTx-plasma, or the corresponding Ringer’s lactate control (RiLac); (**c**,**d**) quantification of the percentage of viable, early and late apoptotic, or necrotic cells at 48 h for (**c**) MiaPaca and (**d**) PaTuS pancreatic cancer cells. Data are presented as mean and are representatives out of three independent experiments.

To analyze whether the decrease in metabolic activity was concomitant with terminal cell death, cells were harvested after control, single, or combination treatment at 48 h. Using DAPI as nuclear counterstain allowing the identification of cells with compromised cellular membranes, and a dye identifying the presence of active caspases within cells, it was possible to distinguish between viable (DAPI^−^, caspase^−^), early apoptotic (DAPI^−^, caspase^+^), late apoptotic (DAPI^+^, caspase^+^) and necrotic (DAPI^+^, caspase^−^) MiaPaca (Figure 3a) and PaTuS (Figure 3b) cells. All treatment regimen reduced the viability of the MiaPaca (Figure 3c) and PaTuS (Figure 3d) cells. The single-agent treatment regimens only modestly reduced the fraction of viable cells, while the reduction was much greater with combination treatment in both cell lines. The most effective regimen in PaTuS was plasma-cisplatin (viability = 76.3%) and cisplatin-plasma (83.3%) followed by gemcitabine-plasma (viability = 76.4%) and plasma-gemcitabine (78.8%) (Figure 3d). In general, responses in MiaPaca cells were greater with plasma-cisplatin (viability = 31.4%) or cisplatin-plasma (37.6%) (Figure 3c). The combination with gemcitabine was weaker compared to that of cisplatin in MiaPaca cells with viability decreasing to 64.7% with plasma-CTx, followed by CTx-plasma.

### 2.4. Combination Therapy Induced Morphological Alterations and Cell Cycle Arrest in Pancreatic Cancer Cells

**Figure 4:**
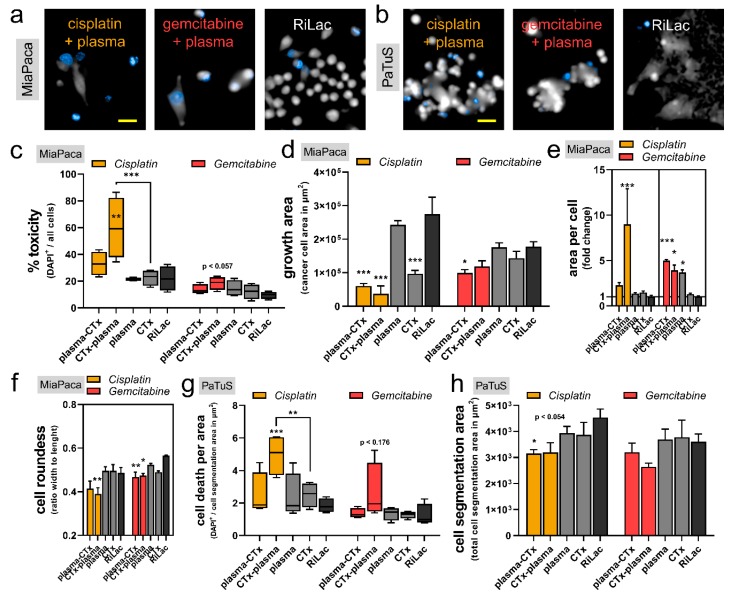
A combination of cisplatin and gemcitabine with physical plasma-conditioned Ringer’s lactate was toxic, reduced the cancer cells’ growth, and altered their morphology. (**a**) Representative images with the DAPI fluorescence channel and digital phase contrast of MiaPaca cells (scale-bar = 50 µm); (**b**) representative images with the DAPI fluorescence channel and digital phase contrast of PaTuS cell (scale-bar = 50 µm); (**c**–**f**) algorithm-based quantification of high-content imaging experiments showing (**c**) the toxicity (% DAPI^+^ events on all events), (**d**) the growth area (area of the pseudo-cytosolic digital phase contrast), (**e**) the area per cell, and (**f**) and roundness of MiaPaca pancreatic cancer cells; (**g**,**h**) algorithm-based quantification of (**g**) cell death (DAPI^+^ events per area) and (**h**) cell segmentation area of treated PaTuS cells. Imaging was performed at 48 h post initial exposure to the treatment liquids. Data are representative out of three independent experiments and are presented as (**c**,**g**) boxplot with their median ± min and max, or (**e**–**h**) as mean + SEM. Statistical significance was calculated utilizing ANOVA.

To further investigate the additive toxicity of the combination treatment on the pancreatic cancer cells, we performed quantitative high content image analysis on several cellular and morphological parameters (Figure 4a,b). To confirm cytotoxicity by fluorescence microscopy, exposing MiaPaca cells to cisplatin first and plasma-conditioned RiLac second led to a substantial and significant elevation of the percentage of dead cells (DAPI^+^/all cell events) (Figure 4c). For all other treatment regimens, cytotoxic responses were observed in tendency. Moreover, the total growth area of MiaPaca cells was also affected by the different treatment regimens at 48 h post initial exposure. In comparison to the RiLac control, both plasma-cisplatin and cisplatin-plasma and also cisplatin alone induced significant growth retardation (Figure 4d). A similar trend was observed when using gemcitabine. The altered cell growth features were accompanied by morphological changes. Individual MiaPaca cells exposed to cisplatin-plasma had an increased area per cell indicative of cellular swelling (Figure 4e). The swelling was also observed with the combination treatment using gemcitabine. As a second morphological feature, the individual cell’s roundness significantly decreased in all combination treatment regimens in MiaPaca cells (Figure 4f). PaTuS cancer cells grow in larger aggregates, requiring different algorithms and quantitative techniques to investigate changes by microscopy. Cell death per growth area was calculated (DAPI^+^/area), and similarly to MiaPaca cells, cisplatin-plasma significantly increased toxicity in PaTuS cells (Figure 4g). Analyzing the growth characteristics of PaTuS cells following the different treatment regimens, significant declines were observed for plasma-cisplatin when compared to the RiLac control (Figure 4h).

**Figure 5:**
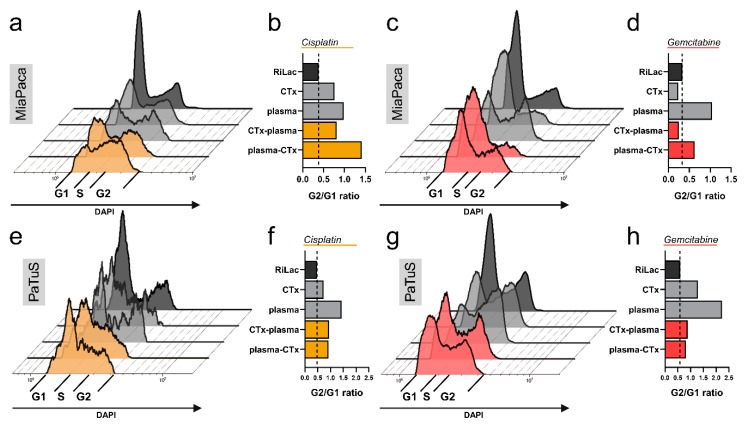
Plasma-conditioned Ringer’s lactate induced cell cycle arrest in pancreatic cancer cells alone or in combination with chemotherapy. (**a**,**c**,**e**,**g**) Representative overlays of flow cytometry data of DNA content, showing the amount of treated pancreatic cancer cells in the G1, S, and G2 phase of the cell cycle; (**b**,**d**,**f**,**h**) quantification of cells in cell cycle arrest (G2/G1 ratio) for MiaPaca cells in treatment regimen with (**b**) cisplatin or (**d**) gemcitabine, as well as PaTuS cells after exposure to respective treatment regimens with (**f**) cisplatin and (**h**) gemcitabine 48 h post-treatment. Data show the mean and are representatives from three independent experiments.

Different growth properties and morphological alterations can be indicative of cellular senescence that is induced by an arrest of the cell cycle. To address this question, the content of nucleic acid inside the cells was quantified by flow cytometry, and the ratio of cells in the G2 over the G1 phase was calculated. The single treatment with physical plasma-conditioned RiLac introduced the most drastic changes in cell cycle arrest, except for the treatment with plasma-cisplatin in MiaPaca cells (Figure 5a–h). However, also drug mono treatment (except gemcitabine in MiaPaca cells) elevate the number of the cells stuck in the G2 fraction. In addition, all combination therapies, with the exception of gemcitabine-plasma in Miapaca cells, showed elevated cell cycle arrest compared to the RiLac control (Figure 5a–h).

### 2.5. Drug Concentrations in Pancreatic Cancer Cells Changed in Combination Treatment as Analyzed Using Mass Spectrometry

**Figure 6:**
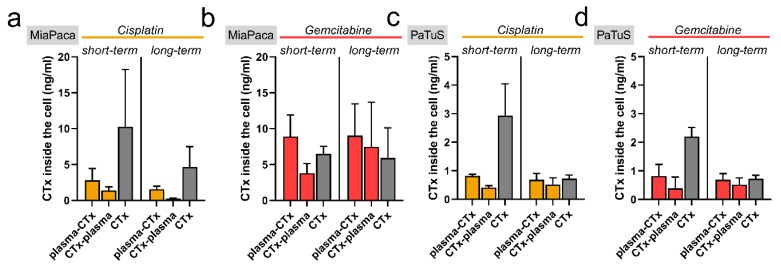
Initial cisplatin and gemcitabine uptake to pancreatic cancer cells were most effective in mono treatments but declined over time. (**a**,**d**) mass spectrometry quantification of the amount of the cisplatin and gemcitabine taken up to MiaPaca (**a**,**b**) and PaTuS (**c**,**d**) pancreatic cancer cells at short (0.5 h) and long-term (24 h). Data are mean + SEM from three independent experiments.

Next, we asked whether the exposition to plasma-conditioned RiLac alters the drug uptake and hence, the intracellular concentration of chemotherapeutic agents. To distinguish between acute effects, e.g., higher membrane penetrability (short-term), and prolonged effects, e.g., differences in expression or function of membrane transporter (long-term), the second treatment was performed with a latency of either 30 min or 24 h. First, it was found that the intracellular drug levels were higher in MiaPaca compared to PaTuS cells (Figure 6a–d). Secondly, short-term concentrations were always higher than long-term concentrations. Third, intracellular levels of cisplatin were generally above those of gemcitabine. These findings directly correlate with the toxic effects that were observed in our cytotoxicity studies. Plasma-treated RiLac reduced intracellular drug levels in MiaPaca cells, regardless of the sequence of combination therapy. For PaTuS, a similar effect was observed in the short-term conditions, while long-term conditions showed roughly equal levels in mono and combination treatments. This points to altered drug uptake or efflux from pancreatic cancer cells exposed to plasma-treated RiLac.

### 2.6. Combination Treatment Abrogated Cancer Growth in an in Ovo 3D-Tumor Model

**Figure 7:**
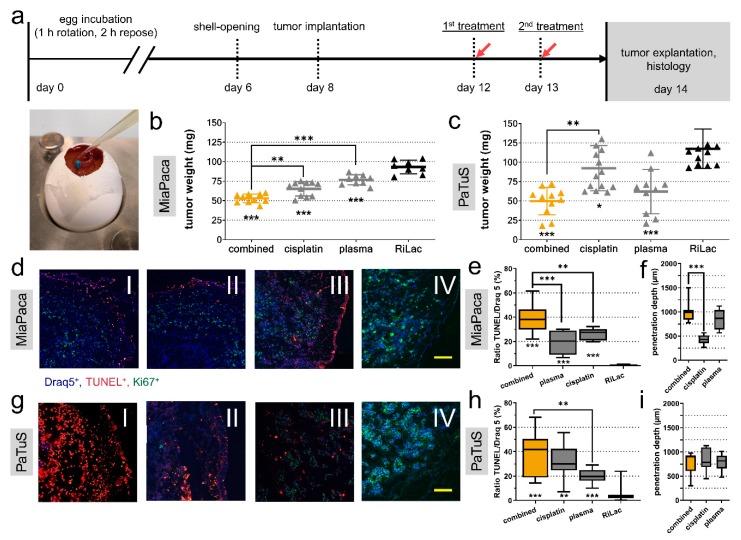
The combination of cisplatin with physical plasma-conditioned Ringer’s lactate showed additive toxicity in three-dimensional tumors grown on chicken embryos. (**a**) Schematic overview of in ovo experiments and image of treatment with liquids of three-dimensional pancreatic tumors grown on the chorion-allantois membrane of fertilized chicken eggs (TUM-CAM); (**b**,**c**) tumor weight quantified at 48 h post-treatment with Ringer’s lactate (RiLac), plasma-conditioned RiLac, cisplatin, or the combination of physical plasma-conditioned RiLac and cisplatin in MiaPaca (**b**) and PaTuS (**c**) tumors; (**d**,**g**) representative images of apoptotic MiaPaca (**d**) and PaTuS (**g**) cells (blue = Draq5^+^, red = TUNEL^+^, green = Ki67^+^; scale-bar = 100 µm) in cryo-sections of the tumors exposed to the combination treatment (I), cisplatin (II), plasma-RiLac (III) and RiLac alone (IV); (**e**,**h**) algorithm-based quantitative image analysis of the percentage of apoptotic cells (TUNEL^+^/Draq5^+^) in MiaPaca (**e**) and PaTuS (**h**) tumors; (**f**,**i**) penetration depth of the treatment regimen in MiaPaca (**f**) and PaTuS (**i**) tumors. Data are representative from two independent experiments with eight to thirteen eggs per group and show (**b**,**c**) individual values with their mean ± SD, and (**e**,**f**,**h**,**i**) median and min to max. Statistical significance was calculated utilizing ANOVA.

To validate our findings in a more realistic model, MiaPaca and PaTuS pancreatic cancer cells were seeded on the chorion allantois membrane of fertilized chicken eggs (TUM-CAM model). This model allows for the growth of three-dimensional tumors (Figure 7a). While these tumors are void of immune cells, they become vascularized to form solid 3D-tumors. Treatment regimens were RiLac alone, plasma-conditioned RiLac, cisplatin alone, or cisplatin in combination with physical plasma-conditioned RiLac. All treatments were applied twice. It was waived to use gemcitabine in a combinational regimen on this in ovo model because cisplatin was identified to be the most promising candidate. The tumors were explanted 48 h after initial exposure to the liquids and weighted. For MiaPaca tumors, all treatment regimen significantly reduced the tumors mass compared to the RiLac control (93.3 ± 8.8 mg) (Figure 7b). Most tremendous changes were observed for the combination treatment with cisplatin and plasma that reduced the tumor mass to 52.9 mg (± 5.6 mg), which was significantly lower than the cisplatin (65.2 ± 9.1 mg) and control (76.8 ± 6.6 mg) treatment (Figure 7b). Control PaTuS tumors were 105.3 mg (± 38.6 mg). Combination treatment significantly retarded tumor growth to 49.8 mg (± 17.9 mg). Cisplatin treatment gave 92.3 mg (± 29.2 mg), while plasma-treated RiLac achieved a significant reduction to 62.1 mg (± 28.6 mg) (Figure 7c). The ability to induce apoptosis is a crucial factor in the evaluation of oncologic strategies. Therefore, we examined the tumors by immunofluorescence for apoptosis by quantifying the amount of TUNEL^+^ cells over all cells (TUNEL^+^/DRAQ5^+^). In MiaPaca tumors, spontaneous apoptosis was a rare event (0.6 ± 0.7%). Treatment with plasma (19.2 ± 9.7%) or cisplatin (26.2 ± 4.6%) significantly increased the rate of apoptotic cells. Combination treatment gave the strongest apoptotic response (38.8 ± 12.1%) (Figure 7e). Next, we quantified the distance of apoptotic cells from the outer tumor layer and found a mean activity depth of 423 µm (± 100 µm) with cisplatin (Figure 7f). However, both, plasma (851 ± 203.4 µm) and the combination treatment (1003 ± 222 µm) significantly showed significantly deeper penetration into the tissue (Figure 7f). In PaTuS tumors, spontaneous apoptosis was observed in 4.3% (± 5.9%) of the tumor cells. However, treatment with plasma (20.0 ± 5.8%) or cisplatin (32.4 ± 12.7%) alone, as well as the combination (37.2 ± 17.9%), significantly increased the rate of apoptosis (Figure 7h). By contrast, differences in penetration depth were not observed (Figure 7i). Furthermore, the percentage of proliferating (Ki-67^+^) cells was calculated. Tumors that received plasma-conditioned RiLac showed decreased proliferation in tendency, but differences were minor with all groups compared to the RiLac control (Supplementary Figure S1h,i).

## 3. Discussion

Most protocols in adjuvant, additive, and palliative CTx for PC are based on gemcitabine either as monotherapy or in combination with, for instance, capecitabine (ESPAC-4 [7]). Despite a better understanding of the pathogenesis of PC, the prognosis has improved only slightly in absolute terms. The best survival rates are observed in resected patients, with a median survival between 13 and 48 months, depending mainly on the tumor stage (i.e., T1/2 or T3/4) [7,8,29]. In metastatic non-resectable pancreatic cancer, median survival is between 5–7.5 months on palliative chemotherapy [30,31]. In this context, best survival rates (11 months) were observed using FOLFIRINOX, a gemcitabine-free scheme; however, its application is limited to those patients in good condition [32]. Therefore, gemcitabine is still the standard for most patients suffering from pancreatic PC.

Nevertheless, and mainly due to the unsatisfactory results of chemotherapy, various pharmacological combination treatment schemes have been investigated in recent years, with usually only a slightly positive effect on survival coming at the costs of a significant increase in treatment-related toxicity [33]. One combination scheme involves gemcitabine and cisplatin, as used in biliary tract cancer [34], bladder carcinoma [35], or bronchial carcinoma [36]. This combination was also analyzed for PC and showed promising results with significantly improved six-month survival and better tumor response. However, no better overall survival could be demonstrated, and the incidence of CTx-related complications was also significantly increased compared to mono-treatment with gemcitabine alone [37].

After transfer into the cell, cisplatin forms adducts with the DNA, and as a consequence, a complex pathway of apoptotic and survival signaling is initiated [38]. Inactivation of cisplatin by intracellular scavengers (e.g., glutathione and metallothionein) is considered to be part of the drug resistance acquired during CTx [39]. Several studies investigated phytochemicals and their potential for sensitizing cancer cells for cisplatin-based CTx [40]. For example, shikonin, a product of a traditional Chinese medicinal plant, increased intracellular ROS concentration in vitro and in vivo and, therefore, enhanced cisplatin toxicity and notably raised selectivity [41].

It has been demonstrated multiple times that cold physical plasma applied either directly or via conditioned liquid acts as an anticancer agent in vitro [42,43,44,45]. Most studies highlight the prominent role of ROS and RNS, as the effects could be abolished by antioxidant scavengers [46]. Due to their high reactivity and usually short half-life, it is challenging to trace single reactive species in liquids or cells and estimate their contribution to biological effects [47,48,49]. In vivo, however, the previously promising in vitro results of plasma-conditioned liquids were reproduced in peritoneal metastatic tumors (gastric cancer [23], ovarian cancer [50], pancreatic cancer [22,51]), recently. These results raise hopes to integrate plasma medicine into modern, multimodal tumor therapies, mainly since the restoration of chemoresistance was observed in glioblastoma cells [18].

Surprisingly, there is little evidence of synergism between plasma treatment and CTx in pancreatic cancer yet. We previously demonstrated an additive effect with gemcitabine and plasma treatment in murine pancreatic cancer cells without additional harm to non-malignant fibroblasts [52]. In vivo, a significant tumor reduction in a murine, orthotopic pancreatic cancer model by the combination of plasma and gemcitabine was observed before [17]. It was also demonstrated that the sequence of administration of plasma and tegafur (a 5-FU prodrug) plays a decisive role between synergism and antagonism [53]. However, these studies were performed with direct plasma treatment and possibly included effects by gas, e.g., plasma-derived electromagnetic fields or UV-radiation. In our current study, we found an additive toxicity of plasma-conditioned RiLac with chemotherapy. This combination therapy was superior to mono-treatment in both cell lines investigated, as demonstrated by reduced metabolic activity and cell viability, and enhanced apoptosis and cell cycle arrest. Additionally, we demonstrated a significant improvement of the anti-cancer capacity of either cisplatin or plasma-conditioned RiLac in an in ovo TUM-CAM model by adding the other modality, respectively.

The first barrier clinically applied substances have to overcome is the cell membrane. ROS/RNS can penetrate or interact with this membrane, as well as use ubiquitous aquaporin channels, whereas cisplatin and gemcitabine enter the cell via different membrane transporters [54,55,56,57]. We hypothesized that plasma-conditioned liquid sensitizes the tumor cell for subsequent CTx via oxidative stress induction. By contrast, we found the intracellular concentration of cisplatin and gemcitabine being reduced in cells conditioned with plasma-treated RiLac. As a possible explanation, we hypothesize that cells exposed to plasma-conditioned RiLac experience oxidative stressed and became inactivated. Hence, the lower levels might have been due to reduced transporter activity in the short-term samples, and an increased portion of terminally dead cells (that nonetheless were still part of the cell pellet investigated by mass spectrometry) with the long-term samples. This hypothesis is supported by the reduction in the metabolic activity of the cells and by other hallmarks of cellular senescence. One of them is the cellular swelling (increased area per cell) that was observed to the greatest extent in our combinational regimen [58,59,60]. Moreover, cell cycle arrest was also observed in the groups that received the plasma-conditioned RiLac, similarly to our previous observations in colorectal cancer cells that had received plasma-conditioned saline [42].

With both cell lines investigated, gemcitabine was inferior compared to cisplatin in terms of cytotoxicity. The combination with plasma-conditioned RiLac was also less effective than that with cisplatin. This may be due to the mechanism of action of gemcitabine, which relies primarily on incorporation into the DNA. However, as we have been able to demonstrate, the plasma treatment leads to a significant increase in cells in the G2 phase, so Gemcitabine is correspondingly less incorporated. In contrast, plasma-derived radicals or cisplatin could reduce the antioxidant capacity of the cell so that the vice versa treatment was correspondingly more effective. This might point towards a more synergistic effect from plasma-conditioned RiLac and cisplatin.

The combination of plasma-conditioned RiLac and CTx was effective in vitro and in ovo. Applications of cytostatic drugs diluted in plasma-conditioned oxidative liquids (such as RiLac) therefore hold promising potential in, e.g., postoperative lavage or HIPEC. From our previous studies using intraperitoneal injections of plasma-conditioned sodium chloride or cell culture medium in mice, we know that plasma-conditioned liquids did not have any observable side effects [22,42]. Especially plasma-conditioned RiLac was previously demonstrated to have a potent anti-tumor capacity [61,62,63]. This was true in vitro and in vivo, and the lactate in RiLac was demonstrated being essential in mediating toxic effects [25,64]. These studies support our findings and hypothesis of such an oxidative liquid being suitable for future clinical applications in combination with standard chemotherapeutics (as cisplatin and gemcitabine) to reduce tumor burden in patients that suffer from, e.g., pancreatic cancer.

## 4. Materials and Methods

### 4.1. Cell Lines and Cultivation

Human pancreatic adenocarcinoma cell lines MiaPaca (MIA PaCa-2; ATCC, Manassas, VA, USA) and PaTuS (PaTu-8988s; DSMZ, Braunschweig, Germany) were used. Cells were maintained in cell culture medium (Dulbecco’s modified Eagle’s medium: DMEM GlutaMAX; Gibco ThermoFisher Scientific, Waltham, MA, USA) supplemented with 10% fetal calf serum, 100 U/mL of penicillin, and 100 µg/mL of streptomycin (all Sigma, Steinheim, Germany). The confluence of cells was controlled via microscopy, and subculturing was performed twice a week, while the passages of the cells were kept below ten. Incubation took place in a cell culture incubator (Binder, Tuttlingen, Germany) at 37 °C and 5% CO_2_ under humidified conditions. Possible mycoplasma contamination of the cell culture was excluded regularly. For the experimental in vitro procedures, the cells were counted using an acoustic-focussing flow cytometer (Attune NxT, ThermoFisher Scientific) and added at a concentration of 1 × 10^4^ cells in 100 µL medium per well in 96-well plates. Alternatively, 1 × 10^5^ cells were seeded in 1000 µL medium in 24-well flatbottom plates (Eppendorf, Hamburg, Germany) for flow cytometry experiments. The therapeutic liquids were scaled up 10× in this setting. Both plate types provide a rim that can be filled with double-distilled water, to prevent excessive evaporation of the cell culture medium in the edge wells.

### 4.2. Physical Plasma and Chemotherapeutic Agents

Cold physical plasma was generated by the atmospheric pressure argon-plasma jet *kINPen* (neoplas tools GmbH, Greifswald, Germany), operated at 1.9–3.2 W and 1.1 MHz, and CE-certified as a medical device class IIa in 2013 [65]. The plasma jet consists of a power supply unit and a handpiece. The latter contains a rod-shaped electrode that excites the argon gas (purity: 99.999%; Air Liquide, Paris, France). Outwardly, the device is shielded by a dielectric capillary. For the plasma-treatment of Ringer’s lactate (RiLac; Hartmann B. Braun, Melsungen, Germany), 100 µL of the liquid was exposed to the plasma of the device running at two standard liters per minute (2 slm). The visible plasma effluent did not directly discharge to the liquid. The distance of the liquid surface to the nozzle (9 mm) as well as treatment times regulated with high precision utilizing a computer-controlled *xyz*-table (CNC step, Geldern, Germany). Immediately after the plasma-conditioning, the plasma-RiLac was transferred to the plate, harboring the pancreatic cancer cells with just priorily aspirated cell culture supernatant. Treatment agents were left for 30 min before replacing them with fresh culture medium. For the detection of H_2_O_2_ in the RiLac, the Amplex UltraRed Assay (ThermoFisher Scientific), and for the detection of nitrate in nitrite, the Griess assay (Cayman Chemical, Ann Arbor, MI, USA) was utilized according to the manufacturers’ instructions. The chemotherapeutic agents cisplatin (1 mg/mL, Teva, Petach Tikwa, Israel) and gemcitabine (1 mg/mL, provided by the University Pharmacy Greifswald, Greifswald, Germany) were stored for a maximum of four weeks at room temperature and protected from light. Following long plasma exposure times, the amount of evaporated liquid was measured via a precision balance (Sartorius, Göttingen, Germany) and was supplemented with double distilled water. Standard dilutions (Table 1) were done in RiLac, and the cells were exposed to the drugs in a similar regimen as for the plasma-RiLac. Oxidation of the hydroxyl radical and peroxynitrite anion indicator hydroxyphenyl fluorescein (HPF, ThermoFisher Scientific) was measured immediately after the treatment of 5 µM of the probe after 120 s of plasma-exposure at the λ_ex_ = 460–490 nm and λ_em_ = 500–550 nm fluorescence channel of a high content imager (Operetta CLS, PerkinElmer, Hamburg, Germany). Measurements of the pH were performed with a pH meter (Mettler Toledo, Columbus, OH, USA).

### 4.3. Metabolic Activity Detection

To asses the metabolic activity of the cancer cells after treatment, they were exposed to 7-hydroxy-3*H*-phenoxazin-3-on-10-oxid (resazurin, Alfa Aesar, Haverhill, MA, USA) at 20 h. The dye can be metabolized by viable cells to generate fluorescent resorufin. Fluorescence was measured after 4 h utilizing a multiplate reader (Tecan F200, Tecan, Männedorf, Switzerland) at λ_ex_ = 560 nm and λ_em_ = 590 nm. The H_2_O_2_ scavenging enzyme catalase (Sigma, Steinheim, Germany) was added at concentrations of 20 µg/mL in some control experiments prior to addition of plasma-conditioned liquid.

### 4.4. Flow cytometry

After incubation, cells were washed with phosphate-buffered saline (PBS; PAN Biotech, Aidenbach, Germany) and detached with accutase (BioLegend, San Diego, CA, USA) containing DAPI (BioLegend) and Caspase 3/7 Green Detection Reagent (ThermoFisher Scientific). The cells were stained for 30 min at 37 °C before washing twice with PBS. Data acquisition was performed with a CytoFLEX *S* flow cytometer (Beckman-Coulter, Brea, CA, USA). For each of the replicates, 10,000 single cells were acquired and analyzed using Kaluza 2.1 analysis software (Beckman-Coulter). For cell cycle analysis, cells were harvested and then incubated with ice-cold ethanol at −20 °C for 1 h. After washing and staining with DAPI, flow cytometry data were analyzed for cell cycle phases using the Michael H. Fox algorithm that is provided within the Kaluza software.

### 4.5. High Content Imaging

Imaging was performed using a high content imager (Operetta CLS). The device operates a high-speed motorized table. Images were acquired using a 20x air objective (NA = 0.4; Zeiss, Jena, Germany) and a 16-bit sCMOS camera with laser-based autofocus. Cells were stained with DAPI in images in the brightfield, digital-phase contrast (DPC), and the λ_ex_ = 535–585 nm and λ_em_ = 430–500 nm fluorescence channels were acquired. Image acquisition settings were kept constant. For each independent experiment, four technical replicates with a total of 36 fields of views per condition and experiment were imaged. For the quantification of cell counts, cell area, and morphology, an algorithm-based analysis was performed using Harmony 4.9 image acquisition and quantification software (PerkinElmer, Hamburg, Germany) after segmenting individual cells via their pseudo-cytosolic DPC signal.

### 4.6. Quantitative Assay for Cisplatin and Gemcitabine

Cisplatin and gemcitabine were quantified in cell pellets after lysis and homogenization of the cells and protein precipitation by adding 500 µL acetonitrile and subsequent centrifugation for 5 min at 14,000 *g* and 4 °C. Ten microliters of the clear supernatant were subjected to liquid chromatography-tandem mass spectrometry (LC-MS/MS) analysis. The analysis was performed with the Agilent 1290 series HPLC system (Agilent Technologies, Waldbronn, Germany) coupled with the QTRAP5500 mass spectrometer (Sciex, Darmstadt, Germany). Chromatography was performed using the analytical column Atlantis^®^ HILIC Silica column, 2.1 × 100 mm (Waters, Milford, CT, USA) by gradient elution with acetonitrile (A) and 0.1% formic acid (B) as mobile phases at a flow rate of 250 µL/min. The applied gradient was as follows: 0–1 min, 99% A; 1–1.1 min, 30% A; 1.1–3 min 30% A; 3–3.1 min 99% A; and 3.1–8 min 99% A. The MS/MS analysis was done in the positive multiple reaction monitoring mode by considering the following mass-to-charge transitions (and collision energies): 302.0/246.0 (12 eV), 302.0/266.0 (20 eV) and 302.0/210.0 (36 eV) for cisplatin and 246.2/111.6 (23 eV), 246.2/95 (60 eV) and 246.2/69 (55 eV) for gemcitabine. The analytical range of the quantitative method for both compounds was between 1–100 ng/mL. During the period of sample analysis, the accuracy of the method was ± 15% (relative error of the nominal values).

### 4.7. Tumor-Chorion-Allantoic Membrane Model (TUM-CAM)

The chorion-allantois membrane tumor model (TUM-CAM) was performed as described in previous studies [66,67]. Briefly, pathogen-free eggs (Valo BioMedia, Osterholz-Scharmbeck, Germany) were incubated for one week at a specialized egg incubator with turning functions (Hemel, Verl, Germany) at 37.5 °C and 65% humidity. On day eight, the eggshells were carefully opened, and a cell suspension (containing 2 × 10^6^ cells in 50 µL matrigel extracellular matrix components; Corning, New York, NY, USA) was added to a sterile silicone ring that was placed on the chorion-allantois membrane (CAM). After a further incubation period of four days, solid tumors with blood vessels sprouting from the CAM have formed inside the ring. On day 12, the treatment was performed. For this, the eggs were randomly assigned to groups and received (I) control RiLac, (II) plasma-conditioned RiLac, (III) cisplatin, or (IV) a combination of plasma-RiLac and cisplatin. RiLac volume was 100 µL of, and the concentrations of the drugs and plasma are given in Table 1. Following treatment, the eggs were restored in the breeder, and on the next day, treatment was repeated. On day 14, tumors were excised and cryo-conservated in liquid nitrogen (−196 °C) embedded in freezing medium (Tissue-Tek O.C.T., Sakura Europe, Alphen aan den Rijn, Netherlands).

### 4.8. Histology

Ultra-thin sections (5 µm) were cut vertically and mounted on microscope slides. Nuclei were counterstained using Draq5 (BioLegend). Apoptotic cells were labeled using the TUNEL assay (In situ cell death detection kit, TMR red; Merck, Darmstadt, Germany) according to manufacturer’s specifications. Proliferating cells were labeled using an anti-Ki67 monoclonal antibody (primary antibody: rabbit anti-Ki67; Bethyl Laboratories, Montgomery, TX, USA) that was marked using a fluorescently labeled secondary antibody (donkey anti-rabbit IgG Brilliant Violet 421; BioLegend)). Microscopy slides were examined using a Keyence *BZ-9000* fluorescence microscope (Keyence, Frankfurt, Germany). Using the software dynamic cell count (BZ-II Analyser, Keyence, Frankfurt, Germany), the ratio of TUNEL positive and negative cells (TUNEL^+^/Draq5^+^ vs. TUNEL^-^/Draq5^+^) was determined [43].

### 4.9. Statistical Analysis

Statistical analysis was performed using Prism 8.3 (GraphPad Software, La Jolla, CA, USA). For statistical comparison between different groups, one-way or two-way analysis of variance (ANOVA II) with *Dunnet’s* post-testing was applied. The levels of significance are displayed as asterisks in the figures (*, **, or *** for the *p*-values <0.05, <0.01, or <0.001, respectively). All experiments were performed at least in three independent runs, and detailed information about the specific number of replicates as well es the presentation of the data (mean/median, min-max, SD/SEM) is given in the figure captions.

## 5. Conclusions

The therapy of pancreatic cancer remains challenging, mainly due to its aggressive and infiltrating growth, e.g., to the peritoneal cavity. Our data suggest that standard anti-cancer chemotherapies of this cavity may benefit from a novel combination therapy using pro-oxidative Ringer’s lactate generated via cold physical plasma. Our approach is elegant and clinically relevant because both the Ringer’s lactate as well as the kINPen used in this study are clinically certified products theoretically ready to be used. Further studies are needed using, for instance, tumor materials from patients ex vivo, or launching individual therapy trials with patients in experimental settings.

## Figures and Tables

**Table 1:** Concentration of adjuvant chemotherapy (CTx) and plasma-conditioned Ringer’s lactate used in this study.

Drug Concentrations or Plasma-Exposure Times to 100 µL of Treatment Liquid for in vitro and in ovo Experiments to Reach at Least IC-25			
Component	Cisplatin	Gemcitabine	Plasma-conditioned Ringer’s Lactate
**MiaPaca**	25 µM	50 µM	60 s/100 µL
**PaTuS**	50 µM	50 µM	120 s/100 µL

## Supplementary Materials

The following are available online at https://www.mdpi.com/2072-6694/12/1/123/s1, Figure S1: Additional information.
