# Supplementary material for: Gas Plasma-Conditioned Ringer’s Lactate Enhances the Cytotoxic Activity of Cisplatin and Gemcitabine in Pancreatic Cancer In Vitro and In Ovo
Source: Cancers (Basel). 2020 Jan 2;12(1):123. doi: 10.3390/cancers12010123 (PMC7017174; doi:10.3390/cancers12010123)
Supplement: Supplementary file 1 [file cancers-12-00123-s001.pdf]

cancers

MDPI

Article

Gas Plasma-Conditioned Ringer's Lactate Enhances the Cytotoxic Activity of Cisplatin and Gemcitabine in Pancreatic Cancer in Vitro and in Ovo

Kim-Rouven Liedtke, Eric Freund, Maraike Hermes, Stefan Oswald, Claus-Dieter Heidecke, Lars-Ivo Partecke and Sander Bekeschus

Supplementary Materials

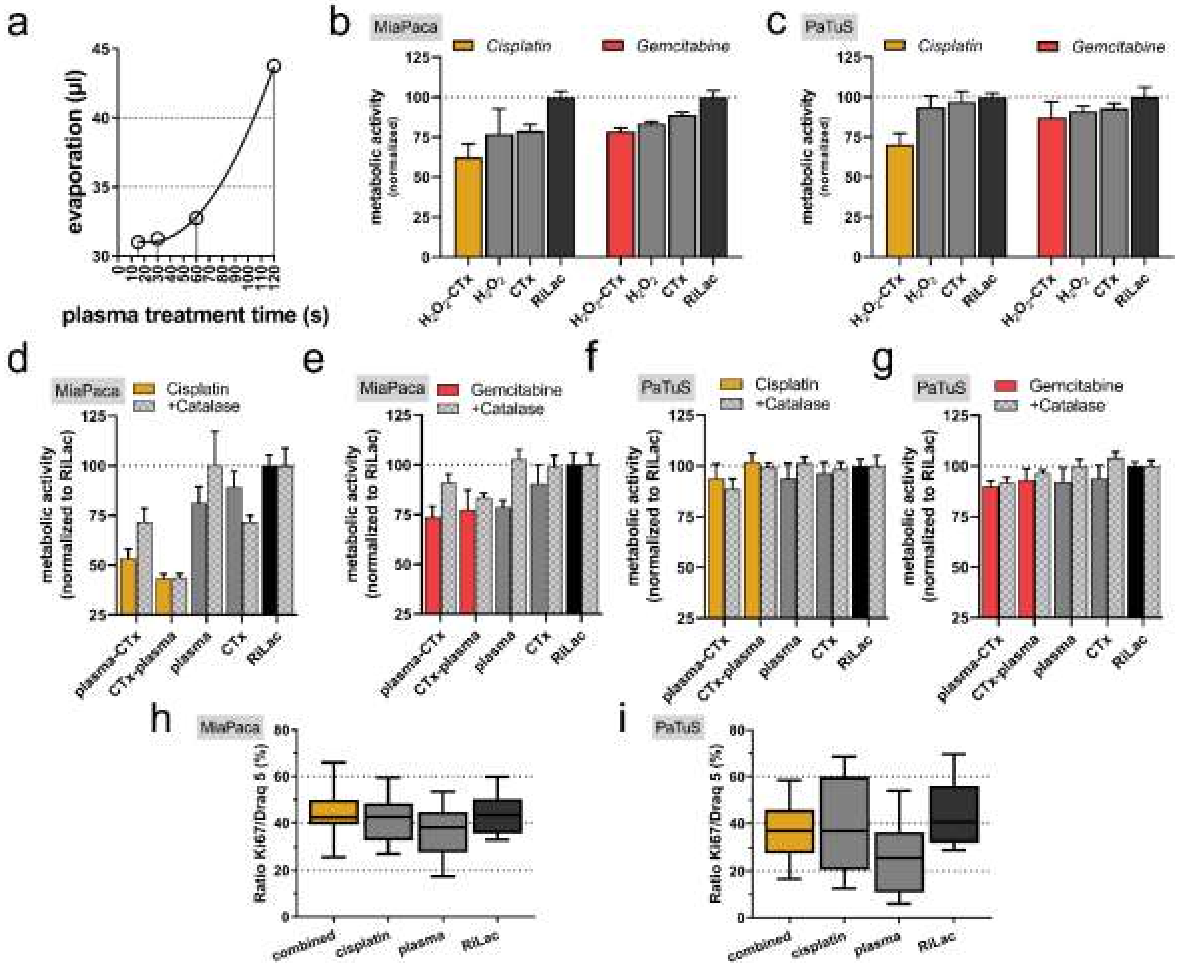

Supplementary Figure 1: Additional information. (**a**) The amount of evaporated liquid of 100 µl plasma-treated *Ringer's* solution; (**b**) the metabolic activity of MiaPaca cells exposed to treatment solutions containing supplemented H_2O_2 instead of plasma-treated *Ringer's*; (**c**) the metabolic activity of PaTuS cells exposed to treatment solutions containing supplemented H_2O_2 instead of plasma-treated RiLac; (**d,e**) the metabolic activity of MiaPaca cells after exposure to treatment solutions with cisplatin and gemcitabine ± catalase; (**f,g**) the metabolic activity of PaTuS cells after exposure to treatment solutions with cisplatin and gemcitabine ± catalase at t = 48 h; (**h**) amount of proliferating cancer cells ($Ki67^+$/$Draq5^+$) in kryo-sections from *in ovo*-grown pancreatic tumors out of MiaPaca cells; (i) amount of proliferating cancer cells ($Ki67^+$/$Draq5^+$) in kryo-sections from *in ovo*-grown pancreatic tumors out of PaTuS cells. Data are (**a–c**) representatives or (**d–g**) out of four independent experiments and are presented as mean + SEM, or (**h,i**) are from independent experiments with between eight and thirteen eggs per group presented as median ± min and max.

© 2020 by the authors. Licensee MDPI, Basel, Switzerland. This article is an open access article distributed under the terms and conditions of the Creative Commons Attribution (CC BY) license (http://creativecommons.org/licenses/by/4.0/).
